# A New Strategy for the Surgical Management of RLN Infiltrated by Well-Differentiated Thyroid Carcinoma

**DOI:** 10.1155/2014/616521

**Published:** 2014-05-14

**Authors:** Jan Boucek, Michal Zabrodsky, Martin Kuchar, Ondrej Fanta, Jiri Skrivan, Jan Betka

**Affiliations:** ^1^Department of Otorhinolaryngology, Head and Neck Surgery, First Faculty of Medicine, Charles University in Prague and University Hospital Motol, V Uvalu 84, 150 06 Prague, Czech Republic; ^2^Department of Anatomy and Biomechanics, Faculty of Physical Education and Sport, Charles University in Prague, Prague, Czech Republic

## Abstract

Well-differentiated thyroid carcinoma (WDTC) represents the most common endocrine malignancy. Despite excellent prognoses exceeding 90% in 10-year follow-up, there are clinically controversial issues. One of these is extrathyroidal tumour extension invading recurrent laryngeal nerve (RLN). The spread outside of the thyroid parenchyma and invasion to the surrounding structures, classified as always T4a, are the most important negative prognostic factor for the WDTC. Conversely, resection of the RLN leads to vocal cord paralysis with hoarseness, possible swallowing problems, and finally decreased quality of life. We propose a new algorithm for intraoperative management based on the MACIS classification, which would allow swift status evaluation pre/intraoperatively and consider a possibility to preserve the infiltrated RLN without compromising an oncological radicality. In the case of a preoperative vocal cord paralysis (VCP) and confirmation of the invasive carcinoma, a resection of the RLN and the nerve graft reconstruction are indicated. Preoperatively, unaffected vocal cord movement and intraoperatively detected RLN infiltration by the invasive WDTC require an individual assessment of the oncological risk by the proposed algorithm. Preservation of the infiltrated RLN is oncologically acceptable only in specific groups of patients of a younger age with a minor size of primary tumour.

## 1. Introduction

Despite rapidly growing global incidence, well-differentiated thyroid carcinoma (WDTC), arising from follicular cells, papillary and follicular, remains one of the most treatable malignancies [1].

Papillary thyroid carcinoma (PTC) is the most common malignancy of the thyroid gland representing about 83% of all thyroid cancers. Prognosis is very good with a ten-year survival up to 98% [1, 2]. However, there is a group of patients with biologically aggressive WDTC, where a relatively high percentage of persistent disease and recurrences affect long-term morbidity and mortality [3]. Molecular alteration associated with the unfavourable prognosis of the WDTC is genetic and epigenetic alterations of signalling pathways—RAS-RAF-MEK-MAPK-ERK pathway (MAPK pathway) and the PI3K-AKT pathway [1]. From a clinical perspective, the most important negative prognostic factor for the WDTC is a spread outside the thyroid parenchyma and invasion to the surrounding structures, classified in TNM classification as always T4a [4, 5]. Ten-year survival drops to 45% compared to patients with the disease restricted to the thyroid gland [6, 7].

Among the surrounding structures, the most commonly affected are strap muscles (53%), recurrent laryngeal nerve (RLN) (47%), trachea (31%), esophagus (21%), and larynx (12%) [8]. The involvement of the aerodigestive tract (non-RLN invasion) is classified according to Shin et al. [9] in five stages to offer surgical guidelines for therapeutic management. In stage I, when the tumor is located entirely within the thyroid gland capsule with no airway invasion, therapeutic recommendation is a total thyroidectomy. In stage II, when the tumor invades the aerodigestive tract perichondrium or is within a close proximity to the muscle but does not invade the cartilage or the muscle deeply, therapeutic recommendation is a complete gross removal by a total thyroidectomy and a partial excision of the cartilage or the muscle. The same therapeutic recommendation is for stage III, when the tumor invades through the airway perichondrium and to the cartilage or to the muscle deeply, but not into the submucosa. In stage IV, the tumor invades through the perichondrium and cartilage or through the muscle and deforms the submucosa but does not penetrate the mucosa and in stage V, there is a gross transmucosal involvement by the carcinoma. A complete resection through the aerodigestive tract is recommended for stages IV and V [9].

Clinically apparent metastatic involvement of the lateral cervical region represents a similarly significant negative prognostic factor as the invasion to the aerodigestive tract, while micrometastases to the central or lateral regions do not affect the prognosis of patients with WDTC [10]. It is shown that a crucial step of the WDTC therapy is a complete surgical removal of the tumor tissue [11, 12] and macroscopically radical resection is the main parameter determining the survival of the patient [13].

## 2. Prognostic Significance of the RLN Involvement

These aspects must be kept in the surgeon's mind considering the management of RLN infiltrated by WDTC. In the context of all mentioned negative prognostic markers, the consideration of the RLN preservation/resection is adequate, only if no other areas, except for the strap muscles, are affected. The surgical radicality remains a subject of debate; the relation to the course of the disease has been established so far only in retrospective studies, not in a large prospective study [7]. Sugitani and Fujimoto demonstrated that patients with the symptomatic papillary microcarcinoma showing preoperative vocal cord paralysis or large node metastasis were likely to die [15]. Also, Ito et al. demonstrated that preoperative vocal cord paralysis may be a sign of a biologically aggressive characteristic; nevertheless, it does not have a significant prognostic value in this study [16].

In particular, the surgical approach in the situation when a WDTC extrathyroid invasion involves only the RLN and a normal vocal cord mobility is preserved remains a controversial issue. Some articles showed no prognostic difference if the affected nerve was either completely resected or when the nerve itself was preserved and the tumor tissue was macroscopically radically removed [13, 17, 18]. However, these studies were retrospective, with a small number of patients and a short follow-up or with nonhomogeneous distribution of extrathyroidal involvement in the compared groups. Nishida et al. did not find any difference in the overall survival, in the number of recurrences, or in the incidence of local, regional, or distant metastases. The study was retrospective and analyzed only with 23 and 27 patients in each group, respectively, and the results were assessed after a relatively short period of follow-up to 6.3 years [18].

Lang et al. retrospectively compared results of 39 and 38 patients, respectively, with the PTC infiltrating the RLN and demonstrated improved short-term and long-term results equally, if the RLN is released from the tumor (by shaving), compared with the radical resection of the infiltrated laryngeal nerve [13]. These two groups were not homogeneous. A significantly higher number of patients with an infiltration of the trachea and other extrathyroidal structures were in the group where the RLN was resected, which considerably impaired oncological results.

“Partial layer resection” constitutes a new, unusual approach when the superficial layer—perineurium—of RLN is resected and the core portion of the nerve is preserved. Functional results were promising, but oncologic results are questionable because of the short follow-up time [19].

Because the intraoperative management of the infiltrated RLN by the WDTC is still controversial issue, we propose a new algorithm based on the MACIS classification [20], which would allow swift evaluation preoperatively and consider the possibility of preserving the infiltrated RLN without compromising oncological radicality.

## 3. WDTC Risk Stratification

For the risk stratification of a particular patient, a number of classifications are used—starting from the international TNM classification (Union Internacional Contra la Cancrum or International Union Against Cancer, American Joint Committee on Cancer—UICC/AJCC) [4], through the AMES classification (age, metastases, extent, and size) [21], the AGES classification (age, grade, extent, and size) [22], and iStage (intraoperative classification according to Ito et al.) [23], finally to the MACIS classification (metastases, age, completeness of excision, invasiveness, and size) [20]. The MACIS classification assesses the completeness or incompleteness of a surgical resection and tumor invasiveness. The MACIS also predicts the development of the WDTC in children and adolescents [24]. A score is obtained by putting values into a formula and a numerical value classifies patient into one of four groups predicting the survival probability (cause-specific survival) in a twenty-year horizon (see Table 1) [5]. The formula is as follows:

(a) for patients less than 40 years old
  (1) Total  MACIS  score=3.1+(0.3×tumor  size  in  cm)+1(if  incomplete  excision)+1(if  locally  invasive)+3(if  distant  metastases);
(b) for patients older or equal to 40 years
  (2) Total  MACIS  score=(0.08×age)+(0.3×tumor  size  in  cm)+1(if  incomplete  excision)+1(if  locally  invasive)  +3(if  distant  metastases).

## 4. Management of the RLN Infiltrated by the WDTC

From a clinical point of view, the surgeon encounters two situations. The first one is a recurrent laryngeal nerve impairment, which is preoperatively manifested by immobility of the vocal cord (VCP—vocal cord paralysis) on the affected side, often accompanied by a hoarseness and eventually swallowing difficulties. The other possibility is a macroscopic infiltration of the RLN nerve exposed during the surgery, with a normal laryngoscopic examination preoperatively. The current recommendation for the first situation with the preoperative RLN palsy is to verify the biological nature of the disease (by a fine needle aspiration biopsy FNAB or intraoperative frozen section) and preserve the nerve only in a situation of a benign lesion or a lymphoma. If an invasive carcinoma is confirmed, the radical resection including a recurrent laryngeal nerve is indicated [14]. An immediate RLN reconstruction with the greater auricular nerve, during the same surgical procedure as the thyroid cancer extirpation, can provide excellent postoperative phonatory function [25]. It was previously considered that the vocal cord immobility resulting from the preoperative malignant infiltration of the RLN is irreversible [26, 27]. The analyses of electrophysiological characteristics of RLN seem to be promising in the estimation of the degree of the tumour invasion and distinguishing the cases in which the intraoperative preserving of infiltrated RLN can lead to functionally satisfactory outcome [26, 27].

When the function of RLN is preoperatively not affected and the nerve involvement is found during the operation, some of the current guidelines recommend intraoperative verification of the biological nature of the disease and preservation of the RLN even in the case of an invasive carcinoma, with a maximum effort to remove all macroscopically apparent portions of the tumour around the nerve [14, 27]. Despite some functional advantages resulting from this approach, the oncological safety remains questionable in the case of reducing the emphasis on surgical radicality. If the RLN infiltration by an invasive carcinoma was detected, a surgeon must consider the deterioration of quality of life due to a resection of the recurrent laryngeal nerve on the one hand and compromising of a course of a malignant disease on the other hand. At the same time, it must be emphasized that the consideration to preserve or resect the RLN is legitimate only if there is no invasion of extrathyroidal structures, except for the well resectable strap muscles. However, it is essential to avoid the injury to both recurrent laryngeal nerves, leading to a bilateral palsy, thus dramatically reducing a quality of a patient's life [14, 28].

**Figure 1:**
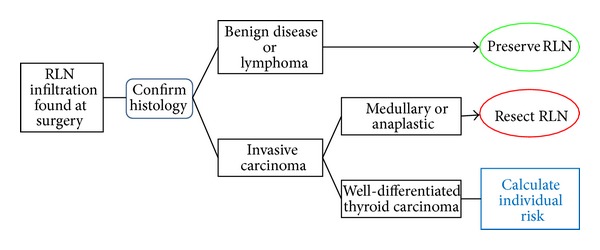
Management algorithm of the RLN infiltration found during the surgery (adapted from Richer and Randolph [14]).

**Figure 2:**
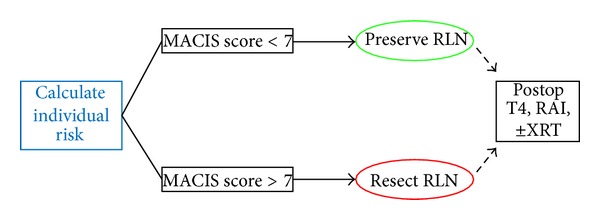
A new algorithm of a surgical management of the RLN infiltration based on the MACIS score.

**Figure 3:**
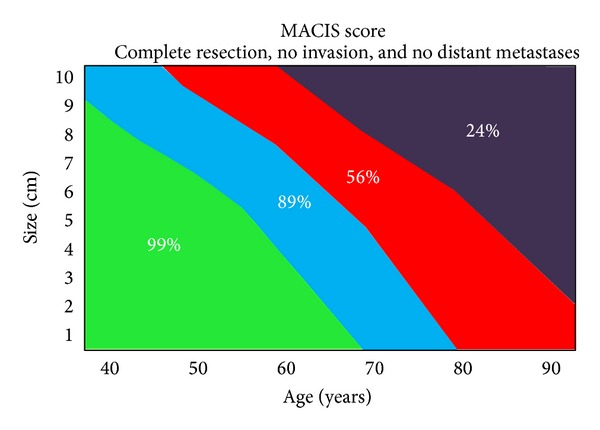
Cause-specific survival according to MACIS classification in case of complete resection, no invasion, and no distant metastases.

**Figure 4:**
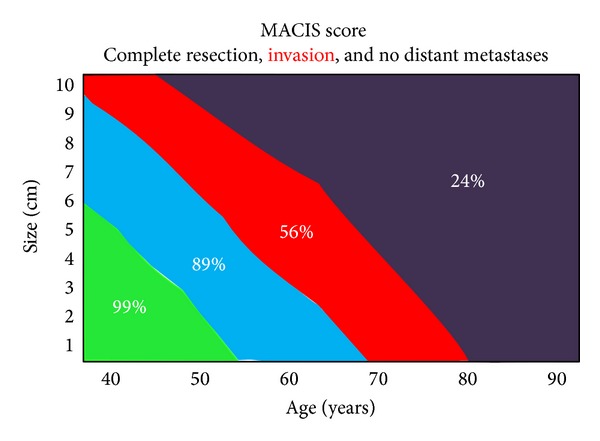
Cause-specific survival according to MACIS classification in case of complete resection, invasion, and no distant metastases.

**Figure 5:**
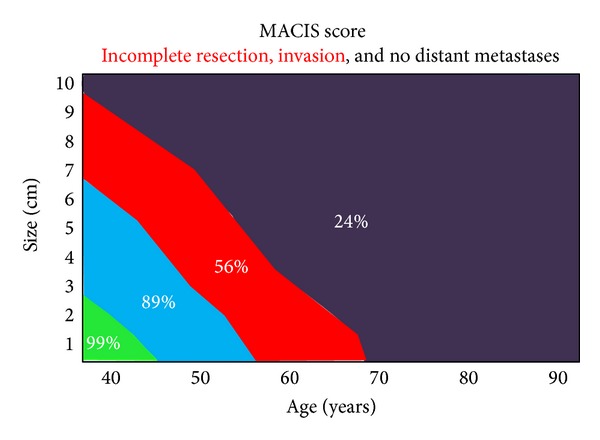
Cause-specific survival according to MACIS classification in case of incomplete resection, invasion, and no distant metastases.

The algorithm in Figures 1 and 2 is a decision-making strategy which guides the surgeon confronted with infiltration of the RLN. It is based on the analysis of a cause-specific survival (CSS) according to the MACIS classification. We consider a score of less than 6.99 as oncologically acceptable, that is, CSS greater than 89% (see Figure 3). When there is no metastatic disease and the infliction of the RLN by the WDTC is removable from the nerve without apparent macroscopic residues, the procedure is oncologically safe only in patients under the age of 50, with a tumour size up to 6 cm (MACIS score = 6.8), under the age of 60 and with the size of the infiltrating tumour up to 3 cm, respectively (MACIS score = 6.7), and under the age of 70, but with only up to 1 cm of the tumour size, respectively, (MACIS score = 6.9) (see Figure 4). However, in the case of maintaining a continuity of the recurrent laryngeal nerve, the tumour residue remains macroscopically visible, the oncological safety is shifted (see Figure 5), and a preservation of the recurrent laryngeal nerve with grossly apparent residuals is acceptable only in patients at the age of 40 and with tumour size up to 5 cm (MACIS score = 6.7) and in patients under the age of 50 and with the tumour size up to 3 cm (MACIS score = 6.9), respectively. In elderly patients or larger tumours, the CSS is significantly reduced; that is, the oncological result of the operation would be unacceptable.

## 5. Molecular Characteristics of the Aggressive WDTC

The advances in a molecular and genetic analysis of the WDTC may bring more accurate decision-making strategies in the future. As mentioned above, molecular alterations associated with aggressive behaviour of the WDTC were identified. The gene mutation of serine/threonine kinase BRAF (BRAF V600E), which is a part of the cascade of the MAP kinase group, plays an important role. The pathological activation of the MAPK cascade then causes upregulation of the protooncogenes and downregulation of the tumour suppressor genes in the cell nucleus [1]. At the same time, it adversely affects the iodine absorption of the follicular cells, thus causing the secondary failure of the radioiodine therapy of the PTC. It was demonstrated that the BRAF V600E is detected in up to 95% of recurrent radioiodine resistant PTC [29, 30]. A clinical correlation of the BRAF V600E mutation with a worse prognosis for patients with PTC, higher aggressiveness in terms of more frequent regional metastases, an increased incidence of recurrence, and a loss of the radioiodine avidity and thus treatment failure has been demonstrated in many studies and meta-analyses [3, 29, 31–33].

Not much is known about the relationship between biological behaviour of the thyroid tumours and the immune system. The possible negative effects of an antitumor immune response by the regulatory T cells (Treg) [34, 35] are considered here as in other localizations [36–38]. French et al. observed significantly increased number of the Foxp3-positive Treg in PTCs showing metastatic disease [35]. A significantly increased number of Treg were also detected by analysis of a FNAB from the lymph nodal PTC metastases, unlike in tumour unaffected nodes [34].

Preoperative knowledge of the presence of these negative prognostic molecular markers would refine the decision-making algorithm in high-risk PTC patients (according to conventional risk stratification schemes). The presence of the BRAF V600E mutation would allow a surgeon to act radically and, in the case of an infiltration, sacrifice the RLN nerve. Conversely, in low-risk PTC cases without any evidence of molecular alterations, a conservative approach and a preservation of the nerve are preferable. The possible precision of a molecular analysis of the tumour microenvironment from preoperative cytological samples (FNAB) is now subjects of an intensive research [31, 39, 40].

## 6. Conclusion

The clinical information about the RLN function and the knowledge of the biological nature of infiltrating tumour is essential for the intraoperative decision-making strategy. In the case of a preoperative VCP and confirmation of the invasive carcinoma, a resection of the RLN and the nerve graft reconstruction are indicated. Preoperatively, unaffected vocal cord movement and intraoperatively detected RLN infiltration by the invasive WDTC require an individual assessment of the oncological risk by the proposed algorithm. Preservation of the infiltrated RLN is oncologically acceptable only in specific groups of patients of a younger age with a minor size of primary tumour.

## Figures and Tables

**Table 1:** MACIS score and survival.

Score	Cause-specific survival (%)
<6	99
6–6.99	89
7–7.99	56
>8	24
